# A heat shock 70kDa protein MaltHSP70-2 contributes to thermal resistance in *Monochamus alternatus* (Coleoptera: Cerambycidae): quantification, localization, and functional analysis

**DOI:** 10.1186/s12864-022-08858-1

**Published:** 2022-09-10

**Authors:** Hui Li, Shouyin Li, Jin Chen, Lulu Dai, Ruixu Chen, Jianren Ye, Dejun Hao

**Affiliations:** 1 Co-Innovation Center for the Sustainable Forestry in Southern China, Nanjing Forestry University, Longpan Road 159, Xuanwu District, Nanjing, 210037 Jiangsu Province China; 2 College of Forestry, Nanjing Forestry University, Nanjing, China; 3 Jiangsu Vocational College of Agriculture and Forestry, Zhenjiang, China

**Keywords:** Global warming, Spermatogenesis, RNA interference, Thermotolerance

## Abstract

**Background:**

Heat Shock Proteins 70 (HSP70s) in insects act on a diverse range of substrates to assist with overcoming extreme high temperatures. MaltHSP70-2, a member of HSP70s, has been characterized to involve in the thermotolerance of *Monochamus alternatus in vitro*, while quantification and localization of MaltHSP70-2 in various tissues and its functional analysis *in vivo* remain unclear.

**Results:**

In this study, temporal expression of *MaltHSP70-2* indicated a long-last inductive effect on *MaltHSP70-2* expression maintained 48 hours after heat shock. *MaltHSP70-2* showed a global response to heat exposure which occurring in various tissues of both males and females. Particularly in the reproductive tissues, we further performed the quantification and localization of MaltHSP70-2 protein using Western Blot and Immunohistochemistry, suggesting that enriched MaltHSP70-2 in the testis (specifically in the primary spermatocyte) must be indispensable to protect the reproductive activities (e.g., spermatogenesis) against high temperatures. Furthermore, silencing *MaltHSP70-2* markedly influenced the expression of other HSP genes and thermotolerance of adults in bioassays, which implied a possible interaction of *MaltHSP70-2* with other HSP genes and its role in thermal resistance of *M. alternatus* adults.

**Conclusions:**

These findings shed new insights into thermo-resistant mechanism of *M. alternatus* to cope with global warming from the perspective of HSP70s functions.

**Supplementary Information:**

The online version contains supplementary material available at 10.1186/s12864-022-08858-1.

## Background

The definition of extreme high temperatures (EHTs) with multiple criteria exists in several research perspectives, such as the temperatures over a given percentile (e.g., the 90th, 95th, or 99th percentile) of temperature distributions for meteorology [1], or exceeding upper physiological thresholds of target organisms for biology [38]. In the current global climate system, there is evidence that a continuing increase in average surface temperatures has considerably increased the frequency and intensity of EHTs [6, 35]. A growing number of attentions have been placed on the effects of EHTs on living organisms, and particularly ectotherms (e.g., insects) [16, 34, 40]. At the individual and population levels, EHTs can disrupt the physiological functions and fitness traits of most insect species, including survival, growth development, and reproduction [8, 15, 54], and eventually cause the decline of insect biomass and/or diversity [7, 10]. Furthermore, recently investigators have reported that climate warming with EHTs as the typical events is a key factor accelerating the outbreak-breakdown cycle of insect populations, which is closely related to the occurrence and dispersal of insect pests [16]. Therefore, studying the comprehensive impacts of EHTs on the individual physiology and population dynamics of insect pests may help to develop strategies for pest control in the context of global warming.

Insects, as small ectotherms, usually establish the physiological responses to heat exposure caused by EHTs, rather than disperse rapidly to track more optimal microclimates [16]. As a well-studied mechanism of heat tolerance, the synthesis and use of heat shock proteins (HSPs) are thought to prevent the denaturing of other physiologically functional proteins under heat exposure. HSP superfamily is grouped into multiple subfamilies, including HSP90, HSP70, HSP60, and sHSP (molecular weights 90, 70, 60, and < 40 kDa, respectively) [18, 45]. Among these subfamilies, the well-characterized roles of HSP70 have been widely described in the insects’ responses to heat exposure [13, 14, 25, 36, 52]. To regulate the formation of protein folding and transport of mature proteins and suppress the aggregate formation under heat stress treatment, HSP70 can bind to client proteins in the early stages of protein folding as molecular chaperones [23, 37]. This chaperone property means a rapid and substantial transcriptional modulation of HSP70 after exposure to high temperatures, which has been demonstrated in many studies [5, 12, 21]. In addition, the roles of HSP70 in thermal resistance have been verified *in vivo* using RNA interference with partial success [19, 33, 50, 51]. Specifically, HSP70 genes knockdown significantly inhibited the feeding behavior, fecundity and survival rate of insects under heat stress. However, the molecular functions of HSP70 in specific tissues in protecting specific substrates or physiological and biological processes (e.g., formation of germ cells and reproductive behavior) are largely unknown. Also, the interaction network between HSP70 and other HSP subfamilies in heat toleration remains elusive [40].

The Japanese pine sawyer *Monochamus alternatus* (Coleoptera: Cerambycidae), an essential global forest pest, causes devastating damage to coniferous trees. Its dispersal range is primarily southern, eastern, and central China belonging to temperate or subtropical zones [17]. This insect pest inevitably suffers from high temperature in summer in its habitat without incurring apparent fitness costs, which is poorly understood. Therefore, the mechanism underlying thermotolerance of *M. alternatus* is a vital topic with implications for integrated management of this insect-disease complex in the context of global warming. Our previous investigations identified a suit of HSP genes in *M. alternatus* larvae induced by a short-term heat shock treatment using comparative transcriptome analysis [29]. HSP70 subfamily, as principal member of these induced HSP genes, was further characterized in different tissues of *M. alternatus* larvae [28]. Among six HSP70 genes of *M. alternatus*, the increased transcripts of *MaltHSP70-2* were at the highest levels upon heat stress, 7109-fold higher than the control levels [28]. Also, recombinant MaltHSP70-2 protein *in vitro* was successfully constructed to verify its stabilized structure and biological activity after heat shock [28]. The ATPase activity of recombinant MaltHSP70-2 protein in *vitro* remained stable at high temperatures, and this recombinant availably enhanced the thermotolerance of *Escherichia coli* [28]. However, quantification and localization of *MaltHSP70-2* in *M. alternatus* adults and functional analysis of *MaltHSP70-2 in vivo* remain largely unexplored.

In this study, we firstly measure the expression level of *MaltHSP70-2* in the whole body of *M. alternatus* adults in the course of heat shock and recovery after heat shock, and tissue-specific distribution of *MaltHSP70-2* in *M. alternatus* adults before and after heat shock was also determined. Subsequently, using Western Blot and Immunofluorescence staining, quantification and localization of MaltHSP70-2 protein in the whole body and reproductive tissue of *M. alternatus* adults were achieved. Finally, we demonstrated the contribution of *MaltHSP70-2* to the thermotolerance of *M. alternatus* and its possible regulatory relationship with other HSP genes using RNA interference. Our findings could improve our understanding of the mechanisms of thermotolerance in *M. alternatus* at the molecular level, and provide a potential target for controlling its population dynamics in the context of global warming.

## Results

### The spatiotemporal dynamics of MaltHSP70-2 gene expression

**Fig. 1:**
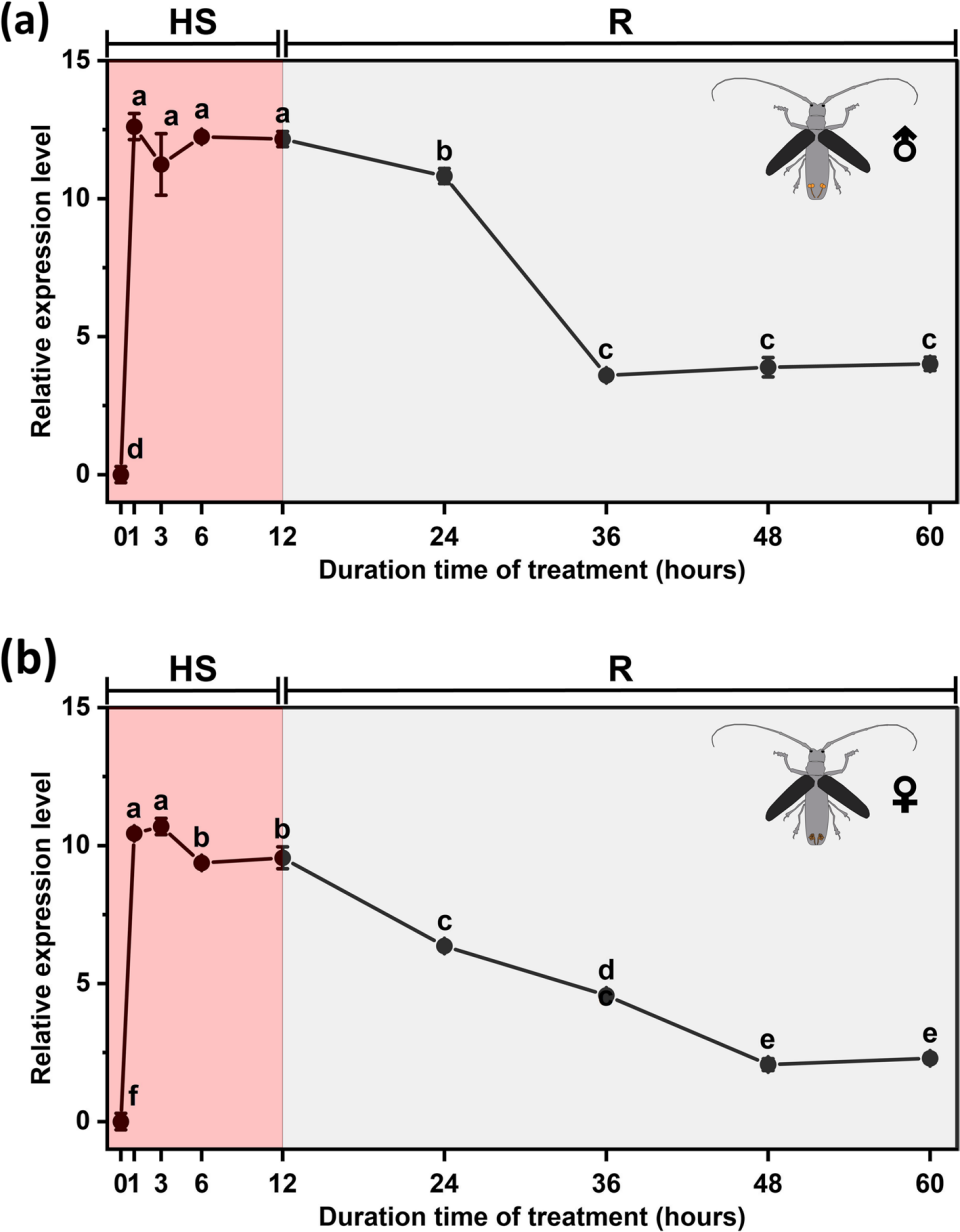
Relative gene expression levels of *MaltHSP70-2* in male (**a**) and female adults (**b**) of *Monochamus alternatus* in the course of heat shock (HS) and recovery after heat shock (R)*.* The data are presented as the mean ± SE (*n* = 5). Different lowercase letters indicate significant differences in the expression level of *MaltHSP70-2* among different treatment times (*P* < 0.05)

The temporal expression pattern of *MaltHSP70-2* under heat stress was shown in Fig 1, and a similar pattern was observed between males (Fig. 1a) and females (Fig. 1b). There was a significant increase in the expression level of *MaltHSP70-2* occurring in the course of both heat shock and recovery after heat shock. There was a bell-shaped relationship between the treatment times and gene expression levels. Specifically, heat shock within a short time (40 °C for 1-3 hours) promptly induced the expression of *MaltHSP70-2* to a peak. As prolonging the time of heat shock (40 °C for 3-12 hours), gene expression of *MaltHSP70-2* stably maintained a high level in males or had a slight decline in females. In the course of recovery after heat shock, the expression level of *MaltHSP70-2* showed a steady decline when compared to heat shock treatments but was significantly higher than the initial level (40 °C for 0 hour).

**Fig. 2:**
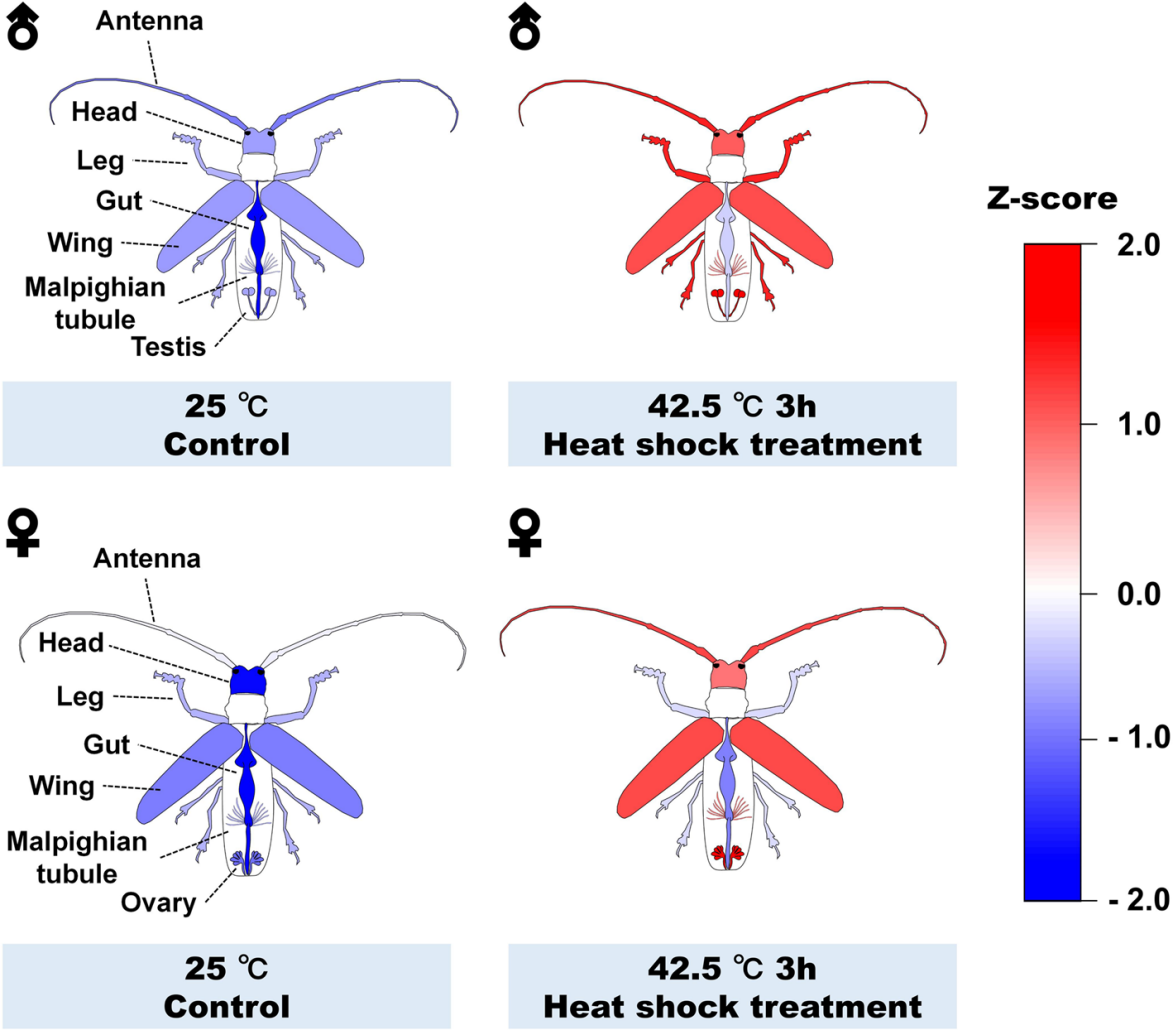
Tissue-specific gene expression of *MaltHSP70-2* after heat shock treatment in male and female adults of *Monochamus alternatus* Red, white and blue layers covering on different tissues indicate high, medium and low expression of *MaltHSP70-2*, repectively. Quantitative data of gene expression levels have been Z-score normalized

The spatial expression pattern of *MaltHSP70-2* gene expression was shown in Fig 2, and a similar pattern was observed between males and females. There was a significant increase in the expression level of *MaltHSP70-2* occurring in all of the tested tissues after heat shock treatment (see Table S2 in detail). Under the ordinary condition (25 °C), *MaltHSP70-2* was observed in all examined tissues and expressed at a very low level in the gut of adults. Under the heat stress condition (42.5 °C for 3 hours), *MaltHSP70-2* was expressed highly in the antenna, head, leg, wing, and malpighian tubule, and notably, extremely high levels of its expression were observed in the reproductive tissues (testis or ovary).

### Western blot analysis of MaltHSP70-2

**Fig. 3:**
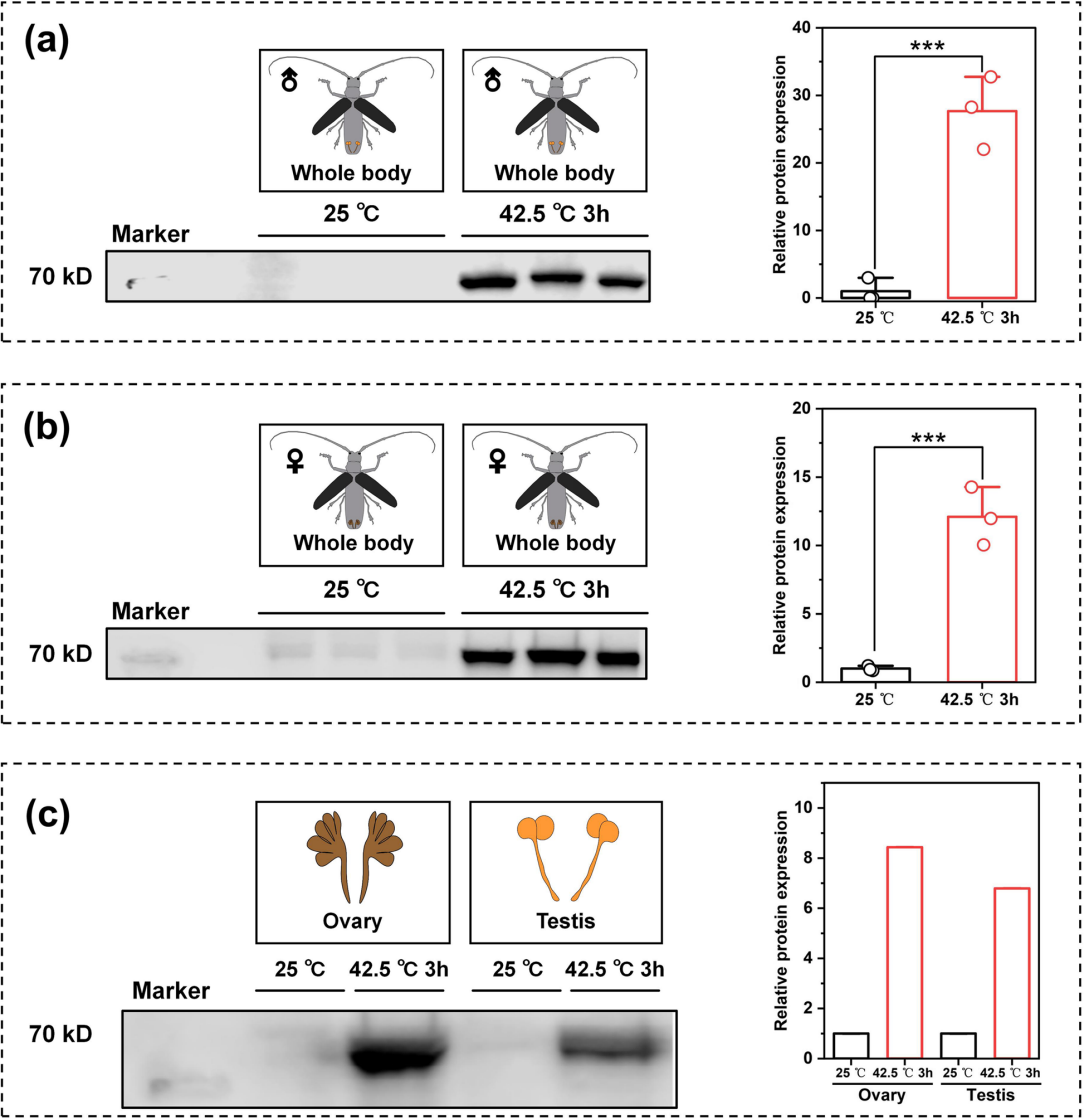
Relative protein expression levels of MaltHSP70-2 after heat shock treatment in *Monochamus alternatus* adults using Western Blot analysis**.** **a**, **b** Specific protein bands and quantification (right panels) of MaltHSP70-2 in the whole body of male and female adults of *Monochamus alternatus* after heat shock treatment. **c** Specific protein bands and quantification (right panels) of MaltHSP70-2 in the reproductive parts of *Monochamus alternatus* after heat shock treatment. The complete picture of protein gels were present in Fig S3. The data of protein expression levels are presented as the mean ± SD and the open circles indicate individual data points (*n* = 3). Asterisks indicate significant differences between control (25 °C) and heat shock treatment (42.5 °C 3h) by independent sample *t*-test (*P < 0.05, **P < 0.01, ***P < 0.001, ns, not significant)

The protein expression levels of MaltHSP70-2 in the whole body and reproductive tissues under the heat stress condition (42.5 °C for 3 hours) are shown in Fig. 3. A stronger band for MaltHSP70-2 around 70 kDa was detected in the crude protein extracted from the whole body of both males (Fig. 3a) and females (Fig. 3b) after heat exposure. The content of MaltHSP70-2 in the males and females was significantly increased by approximately 30-fold and 10-fold, respectively, after heat exposure (*P* < 0.001). A similar increase of MaltHSP70-2 protein occurred in the ovary and testis after heat exposure, while the fold change was approximately 6-fold and 9-fold, respectively (Fig. 3c).

### Immunofluorescence staining of MaltHSP70-2 in testis

**Fig. 4:**
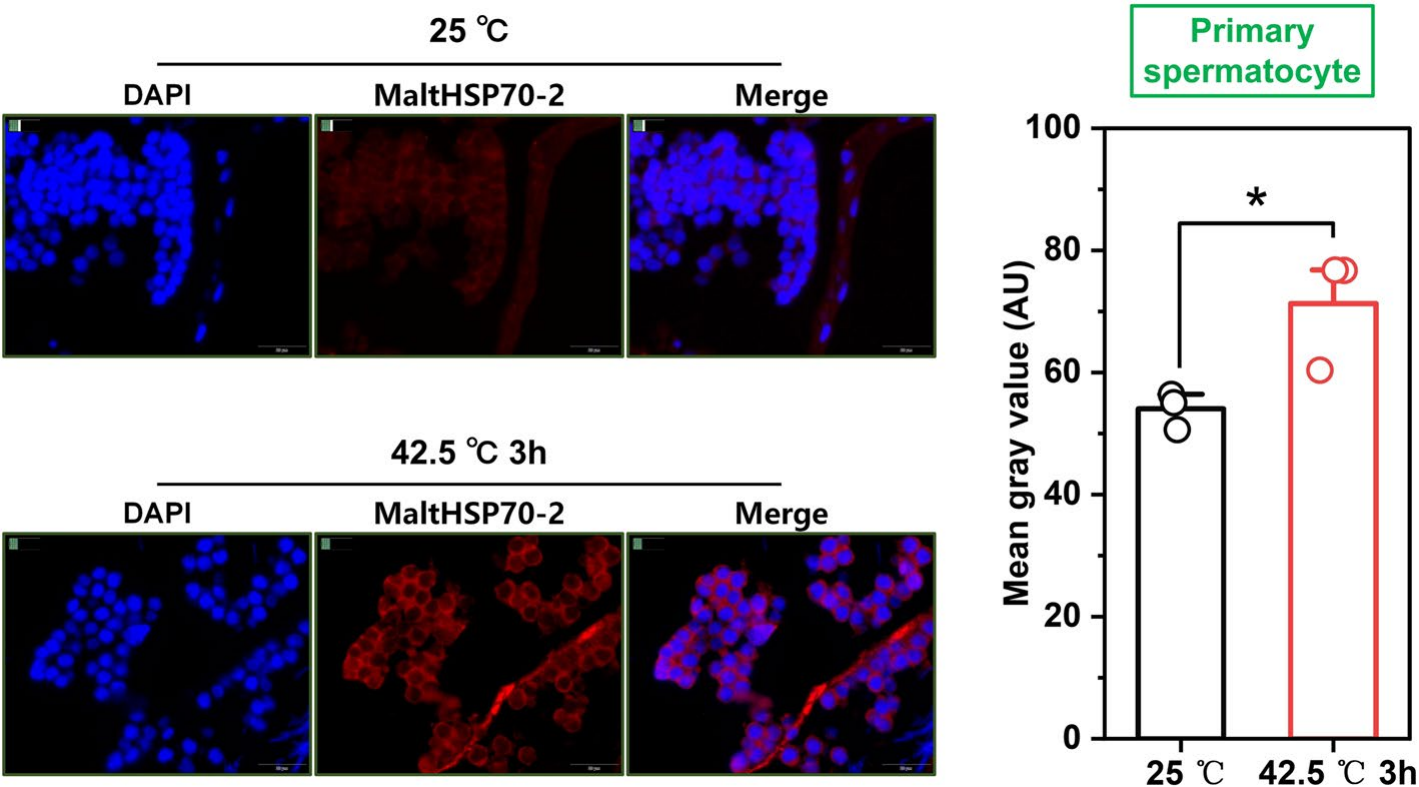
Immunofluorescence staining and quantification (right panels) of MaltHSP70-2 after heat shock treatment in the primary spermatocyte of *Monochamus alternatus*. Gray values are presented as the mean ± SD and the open circles indicate individual data points (n = 3). Significant differences between control (25 °C) and heat shock treatment (42.5 °C 3h) by Student’s *t*-test (*P < 0.05, **P < 0.01, ***P < 0.001, ns, not significant)

**Fig. 5:**
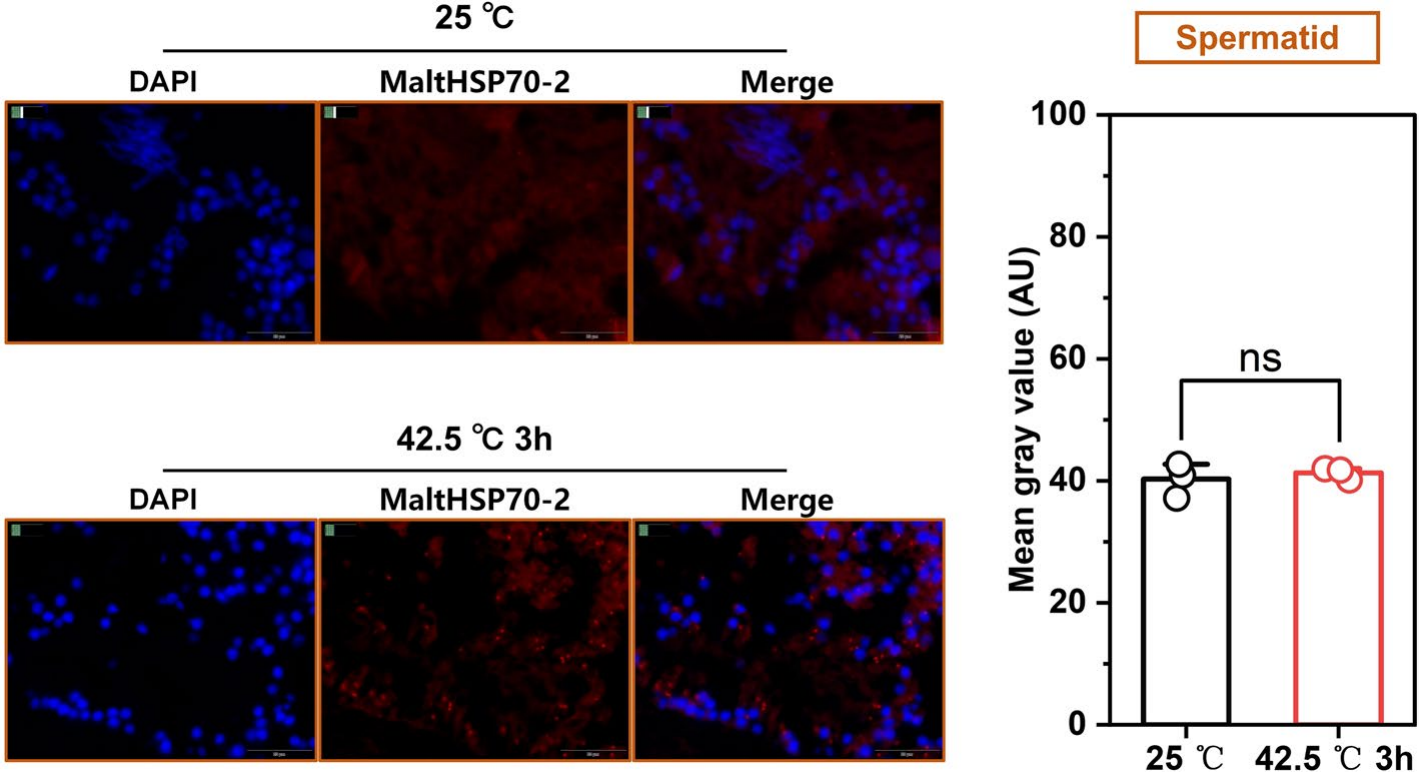
Immunofluorescence staining and quantification (right panels) of MaltHSP70-2 after heat shock treatment in the spermatid of *Monochamus alternatus*. Gray values are presented as the mean ± SD and the open circles indicate individual data points (*n* = 3). Significant differences between control (25 °C) and heat shock treatment (42.5 °C 3h) were determined by Student’s *t*-test (*P < 0.05, **P < 0.01, ***P < 0.001, ns, not significant).

Before immunohistochemical analysis, hematoxylin-eosin staining of *M. alternatus* testis was performed to identify the cell types in the testis. As shown in Figure S1, primary spermatocyte and spermatid are distinguished according to the size of the cells. Spermatid is small, while primary spermatocyte is large. Intracellular localization and semi-quantification of MaltHSP70-2 in the primary spermatocyte (Fig. 4) and spermatid (Fig. 5) were monitored using immunofluorescence staining. We found that an impressive increase of MaltHSP70-2 protein was detected in the cytoplasm of primary spermatocytes after heat exposure (*P* = 0.04), but not in the spermatid (*P* = 0.56) (Figs. 4 and 5).

### Functional analysis of MaltHSP70-2 by RNA interference

To evaluate the role of *MaltHSP70-2* in thermotolerance of *M. alternatus in vivo*, we silenced *MaltHSP70-2* in the males and female adults using RNA interference (RNAi). Silence efficiency of RNAi was measured at different doses and times of double-stranded RNA (dsRNA) injection. 8 μg of dsMaltHSP70-2 was the optimal dose and 3 days post-injection was the optimal effective time (Fig. S2). Under the above condition of RNAi, an approximately 65 % reduction in mRNA levels of *MaltHSP70-2* was observed when compared to the control group (i.e., the green fluorescent protein dsRNA-injected group, dsGFP).

**Fig. 6:**
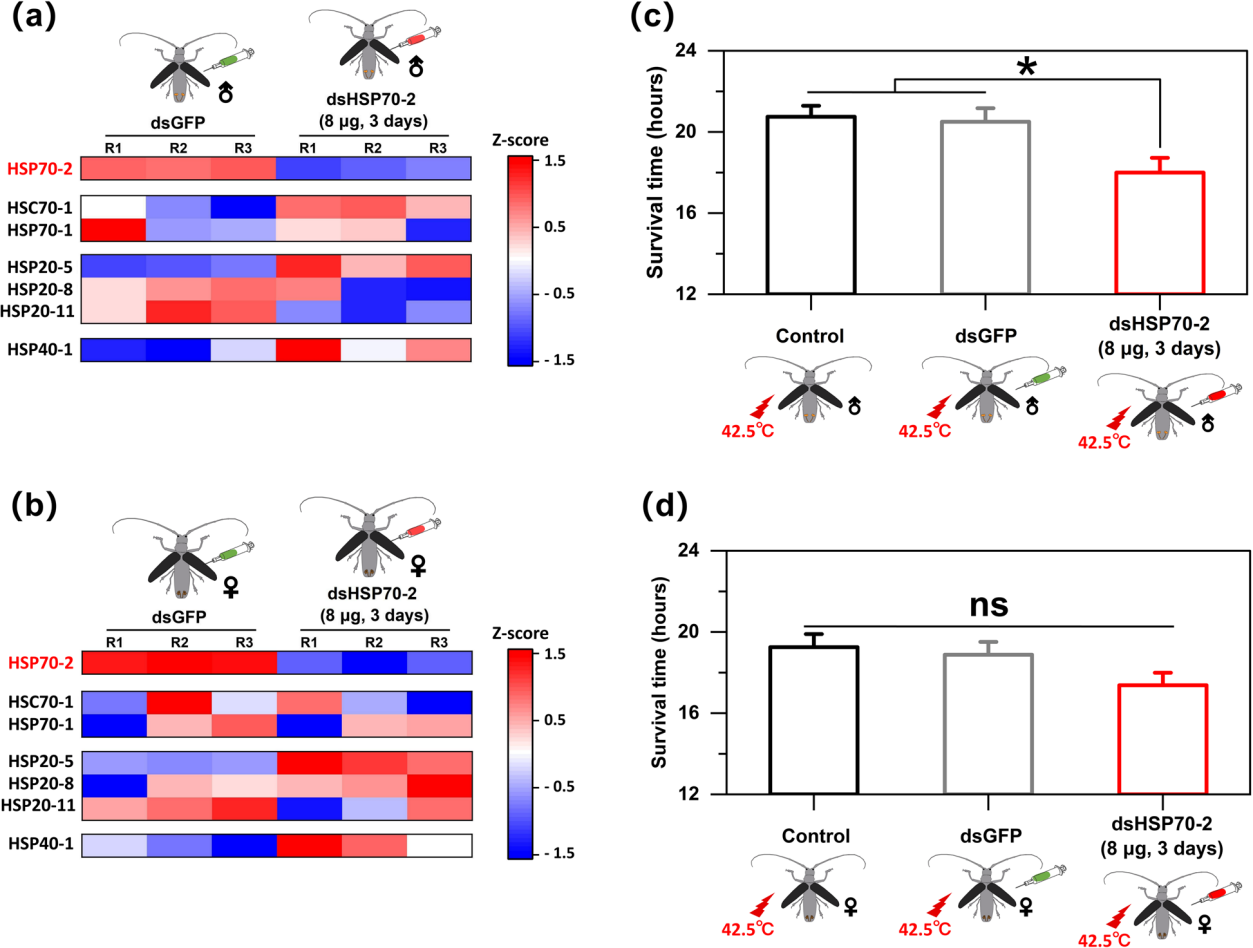
Functional analysis of *MaltHSP70-2* using RNA interference. **a**,** b** Changes in gene expression levels of HSP families in male and female adults of *Monochamus alternatus* when the expression of *MaltHSP70-2* was knockdown. Red and blue bars indicate up-regulated and down-regulated genes in the heatmap, respectively. **c**,** d** Changes in survival times male and female adults of *Monochamus alternatus* after heat shock treatment when the expression of *MaltHSP70-2* was knockdown. Significant differences in survival times between control and dsHSP70-2, dsGFP and dsHSP70-2 were determined by Student’s *t*-test (*P < 0.05, **P < 0.01, ***P < 0.001, ns, not significant)

Effects of *MaltHSP70-2* silencing on other HSP gene expression levels were shown in Fig. 6a & b. For males, expression levels of *HSP20-5*, *HSP40-1*, and *HSC70-1* were significantly up-regulated, while *HSP20-8* and *HSP70-1* showed no obvious changes, and *HSP20-11* was significantly down-regulated (see Table S3 in detail). For females, expression levels of *HSP20-5* and *HSP40-1* were significantly up-regulated, while other HSP genes showed no obvious changes (see Table S3 in detail). In bioassays, compared to the blank control and dsGFP treatment, the survival time of males under the condition of continuous heat stress (42.5 °C) was obviously shortened when injecting with dsMaltHSP70-2 (*P* < 0.05) (Fig. 6c). However, only a slight decrease (not statistically significant) was found in the survival time of females exposed to 42.5 °C after silencing of *MaltHSP70-2* (Fig. 6d).

## Discussion

Increases in the frequency and magnitude of extreme high temperatures (ETHs) pose a significant challenge to the fitness of insect species [16]. Thus, adaptations to heat shock are increasingly pivotal for expanding the geographical distribution of insect species (particularly invasive pests) [40]. The HSP70 subfamily, involving the refolding of hydrophobic residue stretches into their native state, is a sensitive indicator in the biological process of heat tolerance [28]. In this work, we used a set of molecular methods to explore the thermotolerance mechanism of *M. alternatus* from the perspective of HSP70 functions.

Many inducible HSP70 genes have been identified in overcoming thermal stress, and their responses are commonly rapid and drastic [2, 20, 24]. A similar pattern of gene induction was observed in *MaltHSP70-2* from male and female adults: heat shock within a short time (40 °C for 1-3 hours) promptly induced the expression of *MaltHSP70-2* to a peak. Furthermore, we emphasized its expression levels in the course of recovery at 25 °C after heat shock. One interesting finding is that *MaltHSP70-2* remained significantly induced when compared to the initial level, although its expression levels showed a steady decline when compared to heat shock treatments (i.e., a bell-shaped relationship between the treatment times and gene expression levels. See Fig. 1). This is in agreement with an earlier observation, which showed that massive transcription of *Pahsp70* gene in the fat body of *Pyrrhocoris apterus* males started already during the heat stimulus, and its mRNA levels returned close to the initial level within 1 day of recovery at 25 °C [24]. These results indicated that the inductive effect of a short-term heat shock treatment on HSP genes might be stable and long-last, which allows the insect to survive better when ETHs occur.

Nevertheless, the opposite phenomenon, where HSP70 expression levels dramatically increase after a short-term heat shock treatment (1 hour) and then decrease sharply after 2 hours of continuous heat exposure, has occurred in some cases [22, 26]. A possible explanation for this is that the longer-term *de novo* transcription of HSP70 mRNA appears unnecessary because the initial expression of HSP70 is sufficient for protecting the insects against heat stress [32]. Overall, we can infer that the response of HSP70 to various temperature regimes may be closely linked to variations in insect species and their thermotolerance. This hypothesis should be further investigated. In addition, another potential topic deserves in-depth studies to investigate the effects of heat exposure with different forms (e.g., abrupt and ecologically relevant gradual exposure to high temperatures) on the HSP70 gene expression (See the methods from [2]).

Tissue-specific expression of HSPs in response to heat shock has a biologically important significance in insects. However, few reports are available about spatial dynamics of HSP gene expression ([31, 32, 43, 46, 47]; Wang et al., 2019). In our work, and induced expression of *MaltHSP70-2* occurring in all of the tested tissues after heat shock treatment indicated that this HSP70 could have a broad or non-specific mode of action. It is worth noting that despite *MaltHSP70-2* expression present in all tested tissues, it was expressed at the lowest level in the gut of adults exposed to both normal and high temperatures. This finding shared a similarity with the results from Lu et al. [32], where the lowest expression of *NIHsp70* existed in the midgut of *Nilaparvata lugens* that was only 1.86 % of its expression in epidermis. It appears that bacterial endosymbionts colonizing mostly in the gut can assist insect hosts in tolerating heat stress [39, 53], and thus extensive synthesis of HSP in the gut may be not urgent compared to that in other tissues. A small HSP gene (*AP-sHSP21*), in contrast, was most notably induced after heat shock with 43 °C in the midgut of *Antheraea pernyi* [31]. This inconsistency in the spatial expression of HSP genes should be further explored. In addition, enrichment of *MaltHSP70-2* in the exoskeleton of adults (including antenna, head, legs and wing) under heat exposure is expected. Specifically speaking, thermoreceptor neurons involved in the perception and processing of thermal signals are mainly located on the brain, arista, antenna, foot, and wing [3, 30, 40], meaning that deployment of *MaltHSP70-2* in these tissues should be prior and massive. As the primary excretory and osmoregulator organ, Malpighian tubules play an important role in toxin metabolism and reabsorption of water [42]. In early studies, the malpighian tubules of *Drosophila melanogaster* larvae synthesized a set of specific HSPs (with a 64-kDa polypeptide) rather than the common HSP70s after a standard heat shock [48]. However, we found the overexpression of *MaltHSP70-2* in the Malpighian tubules after heat shock, which is consistent with the results of other investigators [43, 46].

Given that the pleiotropic roles of HSP in the development of many traits including oogenesis, spermatogenesis, and embryogenesis [9, 41, 49], we investigated the MaltHSP70-2 expression in the reproductive tissues (testis and ovary) at both the mRNA and protein levels. qRT-PCR and Western blot analysis consistently illustrated that the overproduction of MaltHSP70-2 protein occurred in both ovary and testis after heat exposure. Also, immunofluorescence staining showed the intracellular distribution of MaltHSP70-2 protein in the testis and found that the cytoplasm of primary spermatocyte was the leading site of synthesis and accumulation MaltHSP70-2 protein. In a similar study, the expression and intracellular localization of a small HSP (*CcHsp27*) in the reproductive systems of *Ceratitis capitata* indicates that this HSP is located in the nuclei of the primary spermatocyte and actin cone [11]. It can be speculated that a possible role of HSP in the protection of meiosis or the formation and stabilization of actin cones. Chen et al. reported a recent case also reveals that several HSP70s (*NIHSP70s*) are highly expressed in the adult stage, and gonads of *Nilaparvata lugens* these HSP70s play important roles in thermal tolerance, and ovary and embryonic development [5]. These findings suggest that the frequent biological processes occurring in reproductive tissues may be vulnerable to environmental stresses, and consequently, enriched HSP in these specific tissues is indispensable. In the current work, we only demonstrate the overexpression of MaltHSP70-2 in reproductive tissues of *M. alternatus* adults after heat shock. Further work is required to establish the linkage between HSP70 and the development of reproductive systems under heat conditions.

RNA interference (RNAi) is a promising tool for the cellular function of genes [55]. We successfully silenced *MaltHSP70-2* in the males and females using RNAi in this study. *MaltHSP70-2* silencing caused reduced viability in adults under heat exposure, while a significant effect (*P* < 0.05) was only found in male adults. In combination with the results that MaltHSP70-2 protein in males and females was significantly increased by approximately 30-fold and 10-fold, respectively, after heat shock (Fig. 3A & B), we inferred that *MaltHSP70-2* might play a more dominant role in the resistance to thermal stress for male individuals than that of females. We further monitored the relative expression levels of other HSP genes when *MaltHSP70-2* was knocked down. The tested genes were up-regulated or down-regulated to varying degrees indicating a possible interaction of *MaltHSP70-2* with other HSP genes at the transcription level. As found by Chen et al., the expression level of *NlHSC70-5* was significantly down-regulated when *NlHSC70-4* was silenced [5]. In addition, we found that the expression pattern of these HSP genes when *MaltHSP70-2* was silenced differed between males and females, which was possibly associated with differences in heat tolerance between males and females after *MaltHSP70-2* silencing. Overall, RNAi results provided strong evidence supporting the role of *MaltHSP70-2* in the thermal tolerance of adults, but more work should focus on the specific phenotypic analysis of males and females (e.g., oogenesis, spermatogenesis, and embryogenesis) after *MaltHSP70-2* silencing.

## Conclusions

Overall, *MaltHSP70-2* in adults showed higher expression immediately after heat shock within a short time and maintained a high level in the course of recovery after heat shock. Enrichment of *MaltHSP70-2* in the antenna, head, leg, wing, Malpighian tubule, and notably reproductive tissues (testis or ovary), was observed after heat shock. Using Western blot analysis, we demonstrated the overproduction of MaltHSP70-2 protein in the whole body and reproductive tissues of adults under heat stress treatment. Immunohistochemical assay of MaltHSP70-2 protein in testis specifically showed that primary spermatocyte was the leading site for overproduction of MaltHSP70-2 protein rather than spermatid. Furthermore, *MaltHSP70-2* silencing strongly affected the expression patterns of other HSP genes, and increased the sensitivity to heat exposure in male and female adults to some extent. This study established that MaltHSP70-2, a member of HSP70 subfamily, was closely associated with thermotolerance in the important global forest pest *M. alternatus.* Our findings provide a potential target for controlling its population dynamics under global warming.

## Materials and methods

### Insect culture

*Monochamus alternatus* adults used in this study were obtained from a laboratory colony. The colony was initially established from 4th instar larvae of *M. alternatus* in Nanchang city, Jiangxi province, China (28°50′45.6″N, 115°32′55.6″E) in September 2020. The larvae were individually reared on the artificial diet in a plastic cup (4 cm inner diameter × 5 cm height) until emergence [4]. The newly emerged adults fed on the fresh twigs of masson pines (30 cm length) for supplement nutrition lasting approximately 15 days in a plastic box (11.5 cm length × 11.5 cm width × 5 cm height). The sex of the adult is determined by antennal length. Ten pairs of sexually mature adults (sex ratio 1: 1) as a group were maintained in a net cage (35 cm length × 35 cm width × 40 cm height), and were allowed to mate randomly and oviposit on the trunks of Masson pines (10 cm diameter, 30 cm length). Eggs were collected periodically, and kept in Petri dishes (5 cm inner diameter × 1 cm height) with a moist cotton pad for incubation. All insects were kept in an environmental incubator (MTI-201B, Tokyo Rikakikai, Japan) at 25 ± 0.5 °C with a 70 ± 5 % relative humidity and a photoperiod of 14: 10 h (L: D).

### Sample preparation for quantification and localization of MaltHSP70-2 under heat stress conditions

To determine the temporal dynamics of *MaltHSP70-2* gene expression under heat stress conditions, sexually mature males were kept in the environmental incubator at 40 ± 0.5 °C for 0, 1, 3, 6, and 12 hours, and then were recovered at room temperature for 12, 24, 36, and 48 hours. Vigorous males from each time point were selected and frozen in liquid nitrogen for subsequent tests. Five adults per replicate and three replicates per treatment were used in this experiment. The same sample preparation was performed for female adults.

To determine the spatial dynamics of *MaltHSP70-2* gene expression under heat stress conditions, sexually mature males and females kept in the environmental incubator at 42.5 ± 0.5 °C for 3 hours were sampled as the heat stock treatment, and the individuals kept at room temperature were used as a negative control. Five vigorous males and females per replicate and three replicates from each treatment were dissected to obtain various tissues (including antenna, head, leg, gut, wing, malpighian, tubule, and testis or ovary), and frozen in liquid nitrogen for subsequent tests.

For Western blot and immunofluorescence staining of *MaltHSP70-2*, the scheme of sample preparation was the same as that for determining the spatial dynamics of *MaltHSP70-2* gene expression. Only the whole body and reproductive tissues of female and male adults were sampled for Western blot, and the testis of male adults was sampled for immunofluorescence staining.

### Determination of gene expression levels of MaltHSP70-2 under heat stress conditions

Coding sequence of *MaltHSP70-2* (GeneBank ID: 895064) has been identified in our previous work [28]*.* The samples for determining the spatiotemporal dynamics of *MaltHSP70-2* gene expression under heat stress conditions were crushed into powder in liquid nitrogen for RNA extraction. According to the manufacturer’s protocol, the total RNA of each sample was isolated using an RNA extraction Kit (Tiangen, China). RNA quantity was tested using a Nanodrop 2000 (Thermo Scientific, Waltham, MA, United States), and RNA integrity was monitored on a 1 % agarose gel. Then, the first-strand cDNA was synthesized using the HiScript III RT SuperMix cDNA Synthesis Kit (Vazyme, China) following the manufacturer’s protocol. 2 μL cDNA with a concentration of 200 ng / μL was used as a template for Real-time quantitative PCR (RT-qPCR). According to the manufacturer’s protocol, RT-qPCR experiments were performed using SYBR Premix Ex Taq II (TaKaRa, Japan) on an Applied Biosystem 7500 Real-Time PCR System (Thermo Fisher, Massachusetts, United States) with 20 μL reaction system. PCR conditions were as follows: 5 min at 95 °C, followed by 40 cycles of 10 s at 95 °C and 40 s at 60 °C, and then a melting curve analysis for continuous fluorescence monitoring. Specific primers were designed using Primer Premier version 5.0 (Supplementary Table S1) and synthesized from Sangon, Shanghai, China. Gene expression levels of *MaltHSP70-2* were determined by the 2^−△△Ct^ method with RPL10 as the housekeeping genes [27].

### Preparation of specific antibodies against MaltHSP70-2 protein

Our previous study described the preparation of recombinant MaltHSP70-2 protein *in vitro* [28]. 200 μg of MaltHSP70-2 protein emulsified with complete Freund's adjuvant (CFA) was subcutaneously injected into two New Zealand White Rabbits for the first two times of immunization (an injection is given every 15 days). Then, 100 μg of MaltHSP70-2 protein emulsified with incomplete Freund's adjuvant (IFA) was injected as described above for the next three times of immunization (an injection is given every 15 days). 10 mL of the rabbits’ blood was collected on days 38, 43, 53, 58, and 69 after the first immunization, respectively. Qualitative analyses of these blood samples (cut-off value > 1 after 1: 4000 dilution) were performed using Enzyme-Linked Immunosorbent Assay (ELISA). Approximately 50 mL of positive blood samples was collected for Protein A purification. According to ELISA experiments, the potency of the purified antibody was over 1: 128, 000, and its concentration was over 1 mg/mL. The purified antibody was stored at -80 °C for Western blot analysis and immunofluorescence staining.

### Western blot analysis of MaltHSP70-2 under heat stress conditions

The total protein of samples for Western blot analysis was extracted using Tissue & Cell Protein Extraction Kit (Epizyme, China). Protein concentration was measured using bicinchoninic acid (BCA) protein assay kit (Beyotime, China) and diluted to 4 μg / μL with aseptic water. 20 μL of each protein sample was used to conduct 7.5% sodium dodecyl sulfate-polyacrylamide gel electrophoresis (SDS-PAGE), and then transformed to polyvinylidene fluoride (PVDF) membrane at 80 V for 2 hours. The PVDF membrane was blocked with 5 % skimmed milk in Tris Buffered Saline with Tween-20 (TBST) for 1 hour at 25 °C, then washed in TBST three times. The membrane was incubated with MaltHSP70-2 antibody (diluted with TBST at a ratio of 1: 200 ) at 4 °C overnight. Subsequently, the membrane was washed in TBST three times again, and incubated with the HRP-conjugated goat anti-rabbit IgG antibody (Beyotime, China) (diluted with TBST at a ratio of 1: 1000) at 25 °C for 1 hour. Finally, the membrane was colored using Clarity Western ECL substrate (Bio-rad, America), and imaged on an Odyssey-Fc imaging system (Gene Company Limited, China).

### Immunofluorescence staining of MaltHSP70-2 in testis under heat stress conditions

Testis samples for immunofluorescence staining were fixed in 4 % paraformaldehyde for 24 hours, then were dehydrated with graded alcohol series and embedded in paraffin. Cross-section of the testis was obtained using RM2245 slicer (LEICA, Germany). To observe the basic morphology of cross-section of the testis, hematocylin-eosin staining was performed according to the methods described in Rosenzweig [44]. The section was deparaffinized, rehydrated, and washed in phosphate-buffered solution (PBS) three times for immunofluorescence staining. Subsequently, the section was blocked with two drops of 3% H_2_O_2_ methanol solution for 10 min at room temperature, then washed in phosphate-buffered solution (PBS) for three times, and blocked with 100 μL of 5 % Bovine Serum Albumin (BSA) for 30 min at room temperature. After finishing the block, the section was incubated with MaltHSP70-2 antibody (diluted with PBS at a ratio of 1: 200 ) at 37 °C for 2 hours. Then the section was washed in PBS three times and incubated with TRITC-conjugated anti-rabbit IgG antibody (Beyotime, China) (diluted with TBST at a ratio of 1: 1000) at 37 °C for 1 hour. Finally, the section was stained using Diamidino-2-phenylindole (DAPI) and imaged on a confocal scanning fluorescence microscope DM2500 (LEICA, Germany). The excitation wavelength for MaltHSP70-2 was 549 nm, and for DAPI was 450 nm.

### RNA interference of MaltHSP70-2 and bioassays

To synthesize double-stranded RNA (dsRNA), a 447 bp fragment of *MaltHSP70-2* (see Appendix 1, the region has no similar sequences with other HSP genes) was amplified using the primer containing T7 RNA polymerase promoter at both ends (see Table S1) with T7 RiboMAX™ Express RNAi System (Promega, USA). The dsRNA of the green fluorescent protein (GFP) gene was used as a negative control. The quantity of dsRNA was measured using a Nanodrop 2000 (Thermo Scientific, Waltham, MA, United States), and the size of dsRNA was monitored on a 1 % agarose gel.

To determine the efficiency of RNA interference (RNAi), two doses of dsMaltHSP70-2 (4 μg and 8 μg) were injected into the intersegmental membrane of each adult, and the control injection was conducted with dsGFP (8 μg). At 1, 3, 5, and 7 days post-injection, the whole bodies of eight vigorous adults (sex ratio 1: 1) from each time point were collected for RNA extraction, cDNA synthesis, and RT-qPCR as described above. According to the results of RNAi efficiency, 8 μg of dsMaltHSP70-2 was the optimal dose and 3 days post-injection was the optimal effective time (Fig. S2). Subsequently, the relative expression levels of other HSP genes were measured using RT-qPCR as described above after silencing *MaltHSP70-2* (silencing condition: 8 μg dose, 3 days post-injection).

For bioassays, twenty-four males and twenty-four females were respectively exposed to 42.5 °C after silencing of *MaltHSP70-2* (silencing condition: 8 μg dose, 3 days post-injection). The survival time of each adult was recorded. The adults were defined as death when there was no muscle response to stimulation with a fine brush. The same amount of adults without injection of dsRNA (group name: control) and with an injection of equal quantity of dsGFP (group name: dsGFP) were used as controls.

### Data analyses

mRNA levels of *MaltHSP70-2* at different stages of heat shock treatment were compared using one-way ANOVA followed by Tukey’s HSD test (*P* < 0.05). Statistically significant differences in other quantitative data were analyzed using one-way ANOVA followed by an independent sample *t*-test (*P* < 0.05). All analyses were conducted in SPSS version 20.0 software (IBM SPSS Statistics, Chicago, IL, United States), and plotted with OriginPro version 9.0 software (OriginLab Inc., Northampton, United Kingdom).

## Supplementary Information

**Additional file 1.**

## Data Availability

The data that support the findings of this study are available from the corresponding author upon reasonable request.
