# Supplementary material for: A heat shock 70kDa protein MaltHSP70-2 contributes to thermal resistance in Monochamus alternatus (Coleoptera: Cerambycidae): quantification, localization, and functional analysis
Source: BMC Genomics. 2022 Sep 10;23:646. doi: 10.1186/s12864-022-08858-1 (PMC9464376; doi:10.1186/s12864-022-08858-1)
Supplement: Supplementary file 1 — Additional file 1. [file 12864_2022_8858_MOESM1_ESM.doc]

**Supplementary Tables:**

Table S1. Information of the primers used in this study

| Primer name | Primer sequences (5’→3’) | Product length (bp) | Application |
| --- | --- | --- | --- |
| RPL10-qF | GACAAACGTTTCAGCGGAAC | 83 | qPCR |
| RPL10-qR | TGCTGTTGATCGCCCAAAAC |
| *MaltHSP70-2*-qF | GCAAGTACGACGATCCGAAG | 82 | qPCR |
| *MaltHSP70-2*-qR | TATCTTGGGTTTCCCGCAGT |
| ds*MaltHSP70-2*-T7-F | ggatcctaatacgactcactatagg  AAAGATGAAGGAAACGGCCG | 472 | DsRNA template amplification |
| ds*MaltHSP70-2*-R | GTTCTTTTGGCTCTCTCAGCGG |
| ds*MaltHSP70-2*-T7-R | ggatcctaatacgactcactatagg  GTTCTTTTGGCTCTCTCAGCGG | 472 | DsRNA template amplification |
| ds*MaltHSP70-2*-F | AAAGATGAAGGAAACGGCCG |
| dsGFP-T7-F | ggatcctaatacgactcactatagg  ATGAGTAAAGGAGAAGAACTTTTC | 739 | DsRNA template amplification |
| dsGFP-R | TTTGTATAGTTCATCCATGCCAT |
| dsGFP-T7-R | ggatcctaatacgactcactatagg  TTTGTATAGTTCATCCATGCCAT | 739 | DsRNA template amplification |
| dsGFP-F | ATGAGTAAAGGAGAAGAACTTTTC |
| ds*MaltHSP70-2*-q-F | GGGGAACTCTACAACCCGTG | 209 | qPCR for  RNAi |
| ds*MaltHSP70-2*-q-R | TAAGTACGGCAGCTTGGACG |
| *MaltHSP20-5*-qF | GAAGCAAGACGAGCATGGTT | 190 | qPCR for  RNAi |
| *MaltHSP20-5*-qR | GTGGCTCGCCAGTTTGAATA |
| *MaltHSP20-8*-qF | ACGTCCAACACTTCAAACCG | 165 | qPCR for  RNAi |
| *MaltHSP20-8*-qR | AGTCTGCTACCATCGCAGTT |
| *MaltHSP20-11*-qF | CGGTAGTTGCCTTCGACAAG | 132 | qPCR for  RNAi |
| *MaltHSP20-11*-qR | TCCGGCTTCTCCTCATGTTT |
| *MaltHSP40-1*-qF | GTTGAGCAATGCCCTACCTG | 123 | qPCR for  RNAi |
| *MaltHSP40-1*-qR | TGATTCTTTCGCCCTGACCT |
| *MaltHSP70-1*-qF | TTCCGAGGAACCCTACAACC | 179 | qPCR for  RNAi |
| *MaltHSP70-1*-qR | TACGGCTTCGTCTGGATTGA |
| *MaltHSC70-1*-qF | AGTCGAGAAGGCGAAGAGAG | 186 | qPCR for  RNAi |
| *MaltHSC70-1*-qR | CTTGTTCATGTCGGCGTCTT |

**Table S2. Significance analysis of gene expression levels of *MaltHSP70-2* in different tissues of *Monochamus alternatus* under heat stress conditions.**

| **Tissues** | **Male** | | | **Female** | | |
| --- | --- | --- | --- | --- | --- | --- |
|  | **t-value** | ***P*-value** | **Sig. (two-tailed)^1^** | **t-value** | ***P*-value** | **Sig. (two-tailed)** |
| Antenna | - 54.247 | 0.001 | *** | - 17.581 | < 0.001 | *** |
| Head | - 73.905 | < 0.001 | *** | - 38.486 | < 0.001 | *** |
| Leg | - 41.605 | < 0.001 | *** | - 3.574 | 0.023 | * |
| Gut | - 53.422 | < 0.001 | *** | - 6.610 | 0.003 | ** |
| Wing | - 75.743 | < 0.001 | *** | - 36.920 | < 0.001 | *** |
| Malpighian  tubule | - 15.892 | < 0.001 | *** | - 22.776 | < 0.001 | *** |
| Testis / Ovary | - 88.791 | < 0.001 | *** | - 55.470 | < 0.001 | *** |

^1^ Significant differences in gene expressions between control and heat shock treatments were determined by Student’s t-test (**P* < 0.05, ***P* < 0.01, ****P* < 0.001, ns, not significant)

**Table S3. Significance analysis of gene expression levels of HSP families in male and female adults of *Monochamus alternatus* when the expression of *MaltHSP70-2* was knockdown**

| **Gene** | **Male** | | | **Female** | | |
| --- | --- | --- | --- | --- | --- | --- |
|  | **t-value** | ***P*-value** | **Sig. (two-tailed)^1^** | **t-value** | ***P*-value** | **Sig. (two-tailed)** |
| HSP70-2 | 16.751 | < 0.001 | *** | 12.658 | < 0.001 | *** |
| HSC70-1 | - 2.915 | 0.043 | * | 0.757 | 0.491 | ns |
| HSP70-1 | 0.655 | 0.548 | ns | 0.792 | 0.473 | ns |
| HSP20-5 | - 7.534 | 0.002 | ** | -8.696 | 0.001 | ** |
| HSP20-8 | 1.667 | 0.171 | ns | -1.685 | 0.167 | ns |
| HSP20-11 | 4.589 | 0.010 | * | 1.735 | 0.158 | ns |
| HSP40-1 | - 2.936 | 0.043 | * | -2.930 | 0.043 | * |

^1^ Significant differences in gene expressions between dsGFP and dsHSP70-2 were determined by Student’s t-test (**P* < 0.05, ***P* < 0.01, ****P* < 0.001, ns, not significant)

**Supplementary Figures:**

**
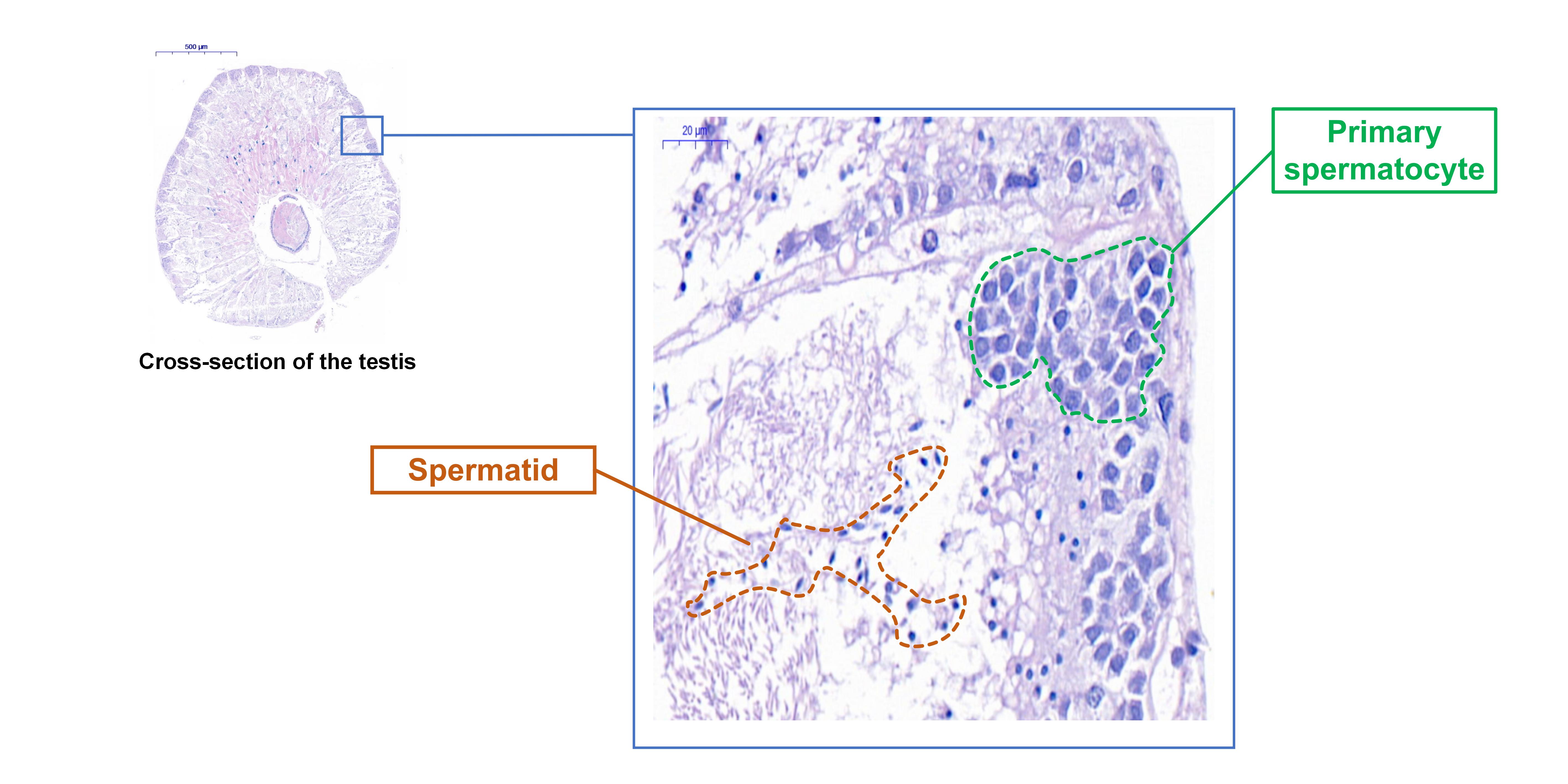
**

**Fig. S1 Cross-section of the testis stained by hematoxylin-eosin.** Primary spermatocyte and spermatid are distinguished according to the size and shape of the cells. Primary spermatocyte is large and subglobose, while spermatid is small and fusiform.

**
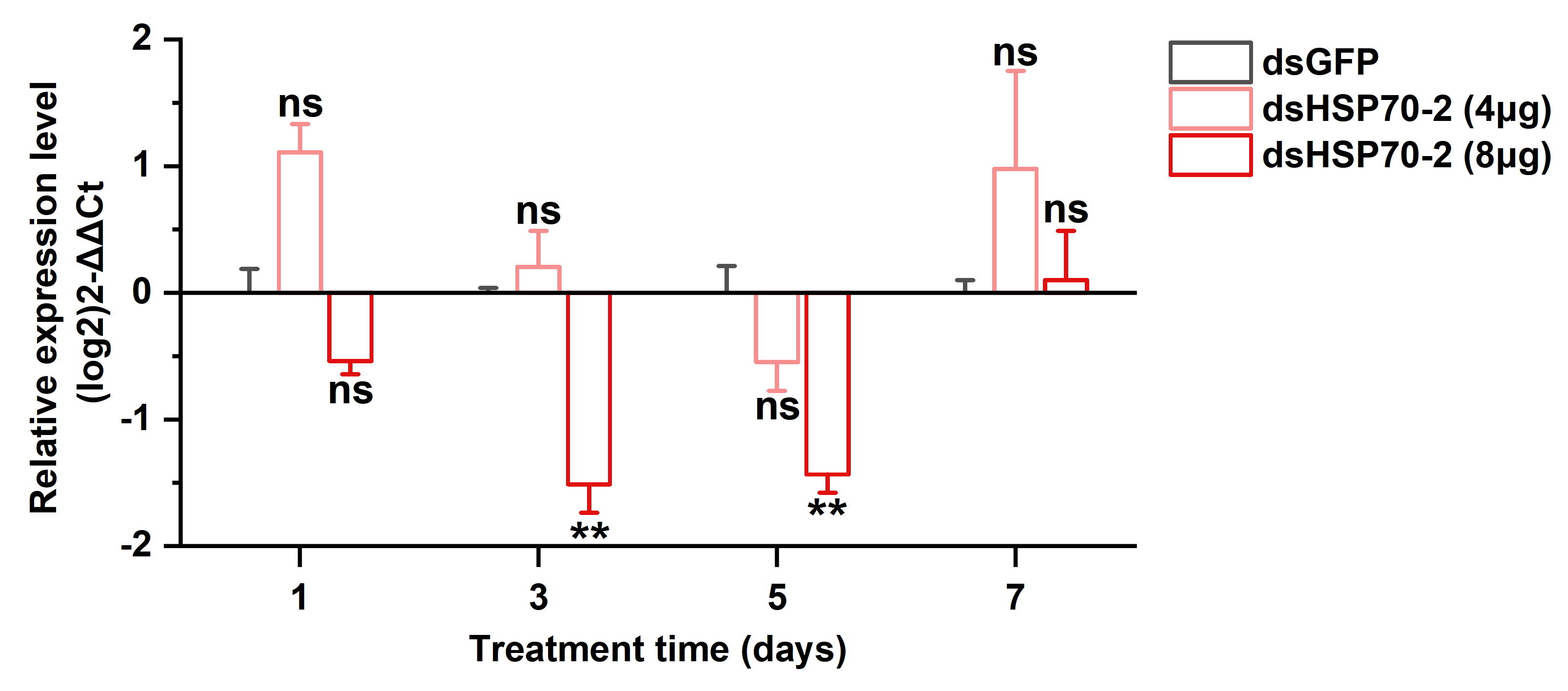
**

**Fig. S2 Efficiency of RNA interference. Significant differences in gene expressions between dsGFP and dsHSP70-2 were determined by Student’s t-test (*P < 0.05, **P < 0.01, ***P < 0.001, ns, not significant).**

**Fig. S3 The complete picture of protein gels for Western Blot analysis.** (a, b) Specific protein bands of MaltHSP70-2 in the whole body of male and female adults of *Monochamus alternatus* after heat shock treatment. (c) Specific protein bands of MaltHSP70-2 in the reproductive parts of *Monochamus alternatus* after heat shock treatment.

**Appendix 1**

**> HSP70-2**

ATGGTAAAAGCTCCAGCAGTTGGCATCGACCTGGGAACCACCTACTCCTGCGTAGGCGTGTGGCAACACGGAAAAGTGGAGATCATCGCCAACGACCAAGGGAATAGAACGACGCCGAGTTATGTTGCCTTTACGGAAACCGAGCGTTTTCTCGGAGACGCTGCTAAGAACCAGGTCGCCATGAACCCCAGCAACACCGTCTTCGACGCTAAGAGGTTGATCGGTCGCAAGTACGACGATCCGAAGATCCAGCAAGACCTCAAGCACTGGCCATTCAAAGTTATCGACGACTGCGGGAAACCCAAGATACAGGTGGAGTATAAAGGAGAAAAGAAGACTTTTTCTCCTGAAGAGATCAGTTCGATGGTGCTGAC**AAAGATGAAGGAAACGGCCGAGGCATATCTGGGGGTACCTGTAAAAGACGCTGTTGTGACAGTCCCTGCGTACTTCAATGATTCCCAAAGACAAGCCACCAAGGATGCAGGCGTAATAGCAGGTCTGAATGTCCTTAGAATAATCAACGAACCAACAGCGGCAGCTTTAGCTTATGGCTTGGACAAAAATTTGAAGGGTGAGAAAAATGTGCTCATCTTCGACTTAGGTGGTGGCACATTTGACGTATCTATTCTTACAATTGATGAAGGTTCGTTGTTTGAAGTGAGATCGACGGCAGGTGACACCCACCTCGGAGGGGAGGACTTTGACAACAGGCTAGTGAACCACCTCGCCGATGAATTCAGGCGTAAATACAAGAAGGACCTTCGAAGCAACCCCAGAGCCCTGAGAAGACTGAGAACTGCCGCTGAGAGAGCCAAAAGAAC**ACTTTCCTCGAGTACCGAAGCCAGCATCGAGATAGACGCCTTATATGATGGTATCGACTATTACACCAAAGTCAGTAGGGCCAGATTTGAAGAACTATGTTCAGATTTATTCAGGGGAACTCTACAACCCGTGGAGAAGGCCCTGATGGACGCTAAAATGGACAAAGGACAAATCCACGATGTGGTCCTTGTGGGAGGTTCCACCAGAATCCCAAAGATCCAACAACTTCTCCAGAATTACTTTAACGGGAAGCCTTTGAACCTGTCCATCAACCCAGACGAAGCCGTAGCTTATGGTGCTGCCGTCCAAGCTGCCGTACTTACCGGAGAAACTGATTCGAAAATCCAGGACGTCCTGTTAGTGGATGTCGCCCCCCTGTCATTGGGTATCGAAACGGCTGGCGGCGTTATGACGAAGATCATCGAACGCAATACAAGAATTCCATGCAAGCAAACTCAAATATTTACGACATATGCCGACAACCAACCGGCAGTTACAGTCCAAGTATTTGAAGGGGAGCGCGCCATGACAAAGGACAACAACCTGCTAGGAACGTTTGATCTCGCTGGAATACCGTTAGCTCCTAGAGGAGTTCCAAAAATAGAAGTAACCTTGGATCTGGACGCTAACGGAATCCTCAACGTATCTGCCAAGGACACCGGCTCCGGAAAGAGCACTAACATCACCATAAAGAACGATAAAGGCAGGTTGTCGCAACAGGACATAGACAGAATGGTGTCCGAAGCTGAACAATACAAGGAAGAAGACGATCGTCAAAGAGAGAGAATTACAGCCCGAAACCAGCTGGAGGGTTACATCTTTCAGCTGAAACAAGCTGTCTCCGACTGTGCCAATAAACTCGGTGCGGAGGACAAAGCTAAAGTTGAAAGTGAATGCGATGACTGCTTGAGATGGCTGGATAACAATTTGTTGGCGGAAAAGGAAGAGTATGAAGAGAAGCAGAGGCAATTGACGAGCGTATGTAGTCCGATTATGGCTAAGTTGTACCAGAATGACCCTCAGAACGGTCAGTTTGGAGACGGTGCTGCTGGTGGAAGTTGTGGTCAGCAGGCAGGAGGGTTTGGAGGTAGTCACGATGGACCAACGATAGAAGAAGTAGACTGA

**Note: bold part is the template of dsRNA synthesis**
